# Awareness and attitude to the law banning smoking in public places in Osun State, Nigeria

**DOI:** 10.1186/1617-9625-12-6

**Published:** 2014-03-27

**Authors:** Samuel Anu Olowookere, Ebenezer Gbenga Adepoju, Olalere Omoyosola Gbolahan

**Affiliations:** 1 Department of Community Health, College of Health Sciences, Obafemi Awolowo University, Ile-Ife, Nigeria; 2 Department of Preventive Medicine, State Specialist Hospital, Asubiaro, Osogbo, Osun-State, Nigeria; 3 Department of Oral and Maxillofacial Surgery, University of Ibadan, Ibadan, Nigeria

**Keywords:** Law on cigarette smoking prohibition, Public places, Osun state, Nigeria

## Abstract

**Objective:**

This study determined the awareness and attitude towards the Osun state prohibition of smoking in public places law.

**Method:**

Descriptive cross-sectional study design. 520 consenting respondents recruited using a convenience sampling method were interviewed using a semi-structured questionnaire covering their smoking pattern, awareness and attitude towards the law of prohibition of smoking in public places in Osun State. Data analyzed using descriptive and chi-square statistics.

**Results:**

Only 38% were aware of the law while none had seen the document. Fifty six percent felt cigarette smoking is a problem that required the law to be implemented, while only 20% agreed that the law will stop tobacco use. The radio (58%), bill boards (45%) and newspapers (44%) were the major sources of awareness of the law. The perception of risk posed to the public and family health by cigarette smoking was poor among the participants.

**Conclusion:**

There is poor awareness and attitude to the law of prohibition of smoking in public places in Osun State. It is necessary to increase sensitization of the general public and enforcement of the law.

## Introduction

There are about 1.3 billion smokers in the world and approximately 80% of them live in the developing countries [1]. Previous studies on the burden of tobacco use in Nigeria had reported a prevalence of current smoking as high as 31.9% in some urban areas and as low as 17.6% in the rural areas [2,3]. A recent study in Osogbo, Osun state reported that 8.7% of their study participants were current smokers [4]. Various studies in Nigeria and beyond have also suggested a high level of passive smoking in the general population [2-5]. For example, Desalu et al. in 2011 reported 38.8% of their study population having regular exposure to second hand smoke with 24.4% from public places [3].

In Nigeria most states do not have a law in place to control smoking in public places [6], and in states where a law exists, such as in Osun state there had been no studies, to our knowledge, that assessed public awareness and attitudes to the law, hence the objective of this study was to determine population awareness and attitudes to the law prohibiting cigarette smoking in public places in Osun state, Nigeria.

## Methods

### Study setting

Osun state was created out of old Oyo state by the Nigerian Federal Government on August 27, 1991. It is located in the heartland of Yoruba people in the South west geo-political zone of Nigeria. It shares the distinctive high urbanization attributes of most parts of Yorubaland. It is bounded in the north by Kwara state, in the east partly by Ekiti state and partly by Ondo state and in the west by Oyo state. According to the 2006 population census, Osun state has a population of 3,416,959. The study site is Osogbo, the capital of Osun state. The population of Osogbo by 2006 census is 288,455. The bill regulating smoking in public places in Osun state was sponsored by the Osun State House of Assembly which was eventually signed into law by the Executive Governor on 14^th^ December, 2009.

### Sample

This is a descriptive cross-sectional study. The required sample size of 384 was calculated using an appropriate statistical formula for estimating the minimum sample size in descriptive health studies [n = Z2pq/d2] and estimated prevalence of cigarette smoking taken as 50% since it is not exactly known. The minimum sample size was increased by 10% to take care of incomplete/non response and refusals. A total of 520 consenting respondents were interviewed out of 640 Osogbo residents (response rate 81.3%) approached to participate in this study. Convenience sampling method was used to enroll these participants during the month of March, 2011. Permission to conduct the study was granted by the Osun State Hospital Ethics and Research Committee. Informed consent was taken from the respondents while they were reassured of the confidentiality of the information obtained.

The respondents included 239 males (46%) and 281 females (54%). Their mean age (SD) was 42.2 (12.4) years ranging from 15 to 68 years. Most of the study participants were civil servants (21.4%) and Transporters (24.8%). They were interviewed with a pretested semi-structured questionnaire that included questions on their sociodemographic information, smoking patterns, awareness and attitude to the law prohibiting cigarette smoking in public places in Osun State, source of awareness of this law and their opinion about cigarette smoking. The respondents’ cigarette smoking status was categorized into ‘ever smokers’ and ‘never smokers’. ‘Ever smokers include former and current smokers. ‘Current smokers’ were respondents who smoked at least a cigarette in the one month prior to the study. ‘Former smokers’ were reported as those who had smoked for six months but had not smoked for at least one month prior to the study. Those who ‘Never smoked’ had never smoked a cigarette [4].

### Statistical analysis

Data was entered into and analysed with SPSS version 16. Frequencies and proportions were used to summarise categorical variables, while quantitative data were presented as means and standard deviations. Chi-square statistics were used to determine the strength of association of different factors with their cigarette smoking status. p values < 0.05 was considered statistically significant.

## Results

A total of 520 respondents were interviewed: 239 males (47%), 281 females (53%), and age range 15 – 68 years (mean 42.2 ± 12.4 years). Majority of the respondents were civil servants (21.4%), Traders (26.5%) and Transporters (24.8%). Most had secondary school education and above (70%) and married (67.4%). Nineteen percent had ever smoked a cigarette with 8.1% current smokers (Table 1).

**Table 1:** Sociodemographic characteristics of the respondents

**Characteristics**	**Frequency (n = 520) N (%)**	
Age group		
19-29	34 (6.6)	
30-39	134 (25.8)	
40-49	152 (29.2)	
50 and above	200 (38.4)	
Sex		
Male	239 (46)	
Female	281 (54)	
Marital status		
Single	150 (28.8)	
Married	350 (67.4)	
Divorced	20 (3.8)	
Highest education level		
None	64 (12.4)	
Primary	92 (17.6)	
Secondary	188 (36.2)	
Tertiary	176 (33.8)	
Occupation		
Civil servant	111 (21.4)	
Traders	138 (26.5)	
Artisan	87 (16.7)	
Transporters	129 (24.8)	
Unemployed	55 (10.6)	
Ethnicity		
Yoruba	466 (89.6)	
Igbo	23 (4.4)	
Others	31 (6.0)	
Religion		
Christianity	294 (56.5)	
Islam	210 (40.4)	
Traditional religion	16 (3.1)	
Smoking pattern		
Current smokers	42 (8.1)	
Former smokers	57 (10.9)	
Never smokers	421 (81.0)	

Table 2 reported the respondents’ awareness and attitude to the Osun state prohibition of smoking in public places. Only 38% were aware of the law prohibiting tobacco smoking in public places in Osun State while none of the respondents had seen the document. Over 28% of ever smokers compared with 40.4% of never smokers were aware of the law (p = 0.026). Also, 15.2% of ever smokers compared with 65.6% of never smokers believe the law is needed to control cigarette smoking (p = 0.001). Fifty six percent felt cigarette smoking is a problem that required the law whereas 20% felt that the law will curb cigarette smoking eventually. The majority (59.6%) of ever smokers compared with 11.9% of never smokers believe smokers deserve right to smoke (p = 0.001). Although 21% felt cigarette smokers have the right to smoke, such right should not infringe on other people’s right to healthy environment.

**Table 2:** Respondents’ awareness and attitude towards the Osun state prohibition of smoking in public places

**Variable**	**Cigarette smoking status**		**Total (n = 520)**	*p value
	**Ever (%)**	**Never (%)**		
	**n = 99**	**n = 421**		
Awareness of the no smoking law				
Yes	28 (28.3)	170 (40.4)	198 (38)	0.026
No	71 (71.7)	251 (59.6)	322 (62)	
Seen the no smoking law document				
Yes	0 (0)	0 (0)	0 (0)	
No	99 (19)	421 (81)	520 (100)	
The law is needed to control smoking				
Yes	15 (15.2)	276 (65.6)	291 (56)	0.001
No	84 (84.8)	145 (34.4)	229 (44)	
Smoking affects public health				
Yes	16 (16.2)	278 (66)	294 (56.5)	0.001
No	83 (83.8)	143 (34)	226 (43.5)	
Law will stop cigarette smoking				
Yes	12 (12)	92 (22)	104 (20)	0.001
No	23 (23)	211(50)	234 (45)	
Don’t know	64 (65)	118 (28)	182 (35)	
Smokers deserve right to smoke				
Yes	59 (59.6)	50 (11.9)	109 (21)	0.001
No	40 (41.4)	371 (88.1)	411 (79)	

*chi-square statistic.

Table 3 reported the respondents’ opinion towards cigarette smoking. Over 15% of ever smokers compared with 64.1% of never smokers agree that smoking is dangerous to health (p = 0.001). 46.5% of ever smokers compared with 29.9% of never smokers stated that smoking at least one cigarette per day is safe (p = 0.002). Also, 32.3% of ever smokers compared with 21.1% of never smokers agree that smokers should not smoke at home (p = 0.018). The majority (56.5%) agreed that smoking affects family health; 33% felt smoking 1 cigarette sticks or more per day is safe. Only 20.4% agreed there should be smoke free areas in public places whereas 23% felt smokers should not smoke at home.

**Table 3:** Respondents’ opinion on cigarette smoking

**Variable**	**Cigarette smoking status**		**Total (%)**	*p value
	**Ever (%)**	**Never (%)**	**N = 520**	
	**n**_**1**_ **= 99**	**n**_**2**_ **= 421**		
Smoking is harmful to health				
Agree	15 (15.2)	271 (64.1)	286 (55)	78.452; 0.001
Disagree	84 (84.8)	150 (35.9)	234 (45)	
Number of cigarette stick safe				
None	53 (53.5)	295 (70.1)	348 (67)	9.901; 0.002
≥1	46 (46.5)	126 (29.9)	172 (33)	
Smoke free environment is necessary				
Agree	26 (26.3)	80 (19)	106 (20.4)	2.603; 0.107
Disagree	73(73.7)	341 (81)	414 (79.6)	
Smokers should not smoke at home				
Agree	32 (32.3)	89 (21.1)	121 (23.2)	5.614; 0.018
Disagree	67 (67.7)	332 (78.9)	399 (76.8)	

*chi-square statistic.

Table 4 reported the respondents sources of awareness of the Osun state prohibition of smoking in public places. The radio (58%), bill boards (45%) and newspapers (44%) were the commonest sources of information about the law in Osun state.

**Table 4:** Sources of awareness of the Osun state prohibition of smoking in public places

*Sources of awareness	**Frequency (n = 198) N (%)**
Radio	115 (58)
Bill board/poster/leaflet	89 (45)
Newspapers	87 (44)
Health workers	73 (37)
Television	69 (35)
Friends	45 (23)
School	26 (13)
Internet	8 (4)
No response	6 (3)

*Multiple responses.

## Discussion

This population based study assessed the awareness and attitude of the respondents to the Osun state prohibition of smoking in public places. It reported low awareness among respondents with the radio as the main source of awareness. It should however been noted that the radio remains the commonest source of general awareness among this population making it a very important route of communication with the populace. Hence policy makers need to make use of such medium to inform, educate and communicate to the general public about this law.

Studies had showed that smoking is the most important avoidable cause of premature morbidity and mortality in the world [1,5]. This study reported about one-fifth of respondents were ever smokers. Several studies in Nigeria had reported similar finding [6].

Over three fifth of the respondents were not aware of the law prohibiting smoking in public places while none of the study participant had read the actual document. This situation can make it difficult for the legislation in public places to be effective therefore, it is essential that there is more sensitization of the general public or else the chances of failure remain high. It is therefore important to disseminate information about the law in order to eliminate smoking in public places. Its proper implementation and enforcement will further reduce tobacco induced diseases in this study environment [5,7].

While the majority felt that the law prohibiting cigarette smoking in public places in Osun State is necessary, fewer smokers saw the necessity of this law in protecting both their and public health. In fact, some smokers felt that the law cannot stop smoking in public places and that smokers deserve the right to smoke wherever they like at anytime. These views expressed by the smoking population contradict the general opinion of non smokers. This further showed the need to enhance awareness and support enforcement of the law. Previous studies had reported that few states in the country have adopted the law, therefore its successful implementation lies on both the government and the citizens; the power to implement and enforce the law lies with the government while the will power to embrace and obey the law lies with the citizens [5].

Some of the smokers in our study believed that cigarette smoking is not dangerous to their health. This view needs consideration as it implies that these smokers are either unknowingly or carelessly endangering their life but that of their close family members, co-workers and neighbours. Also, only a few smokers felt a smoke free environment is necessary. This calls for urgent attention in order to reduce the effects of tobacco exposure among this population. There is urgent need for more public education in Osun state and beyond on the danger of cigarette use in both the smoking and non smoking population.

To the best of our knowledge, this is the first study performed to assess the awareness and attitude of Osogbo residents to the Osun state prohibition of smoking in public places law. We were also unable to identify other studies done to assess such law in operation in other states in Nigeria. A limitation is the use of the convenience sampling technique to select the study population, which limits its generalizability to the population of the State or Nigeria in general. This was done to ensure a proportionate increase in the smoking population among the selected respondents.

In conclusion, the present level of awareness of the law prohibiting cigarette smoking in public places in Osun state was poor. It is recommended that strategies to increase the present level of public awareness on dangers of cigarette smoking and enforcement of the law of prohibition of smoking in public places should be put in place. These strategies will include awareness creation activities in public places, bill boards, jingles on the radio and other mass media venues.

## Competing interest

The authors disclose no potential conflicts of interest.

## Authors’ contributions

AEG and OSA made substantial contributions to conception and design of the study while all the authors were involved in data collection, analysis and interpretation. All authors were involved in writing the manuscript and approved the final copy.

## Short report

There is poor awareness and attitude to the law banning cigarette smoking in public places in Osun state, Nigeria. Also, the perception of risk posed to the public and family health by cigarette smoking was poor. Sensitization of the general public and enforcement of the law is essential.
